# Supplementary material for: Bayesian reconstruction of transmission within outbreaks using genomic variants
Source: PLoS Comput Biol. 2018 Apr 18;14(4):e1006117. doi: 10.1371/journal.pcbi.1006117 (PMC5927459; doi:10.1371/journal.pcbi.1006117)

# Bayesian Reconstruction of Transmission within Outbreaks using Genomic Variants

Nicola De Maio[1,*], Colin J Worby[2], Daniel J Wilson[1,3,‡], Nicole Stoesser[1,‡]

**1 Nuffield Department of Medicine, University of Oxford, Oxford, United Kingdom**
**2 Department of Ecology and Evolutionary Biology, Princeton University, Princeton, USA**
**3 Wellcome Trust Centre for Human Genetics, University of Oxford, Oxford, United Kingdom**

**‡These authors contributed equally to this work.**
*** E-mail: nicola.demaio@ndm.ox.ac.uk**

# Supplementary Text S1

# References

1. Hasegawa M, Kishino H, Yano Ta. Dating of the human-ape splitting by a molecular clock of mitochondrial DNA. J Mol Evol. 1985;22(2):160–174.

2. De Maio N, Schrempf D, Kosiol C. PoMo: an allele frequency-based approach for species tree estimation. Systematic biology. 2015;64(6):1018–1031.

3. De Maio N, Wu CH, Wilson DJ. SCOTTI: efficient reconstruction of transmission within outbreaks with the structured coalescent. PLoS computational biology. 2016;12(9):e1005130.

4. Worby CJ, Lipsitch M, Hanage WP. Shared genomic variants: identification of transmission routes using pathogen deep sequence data. American Journal of Epidemiology. 2015;.

**Figure A. Computational demand of BadTrIP.** Mean computational demand, in seconds, to run $10^5$ MCMC steps (blue) and to achieve an effective sample size of 200 for the posterior probability (grey) in BadTrIP. Each barplot represents the mean over 10 simulations, and the y axis values of each barplot are seconds. The three rows in the table represent the number of hosts in the simulated outbreaks (3, 5 or 10) and the two columns represent different mutation rates ($10^{-5}$ corresponds to the "base" scenario in Figure 3 while $10^{-6}$ corresponds to the "low mutation" scenario in Figure 3). On top of each bar is the same running time but represented in minutes ("m") or hours ("h").

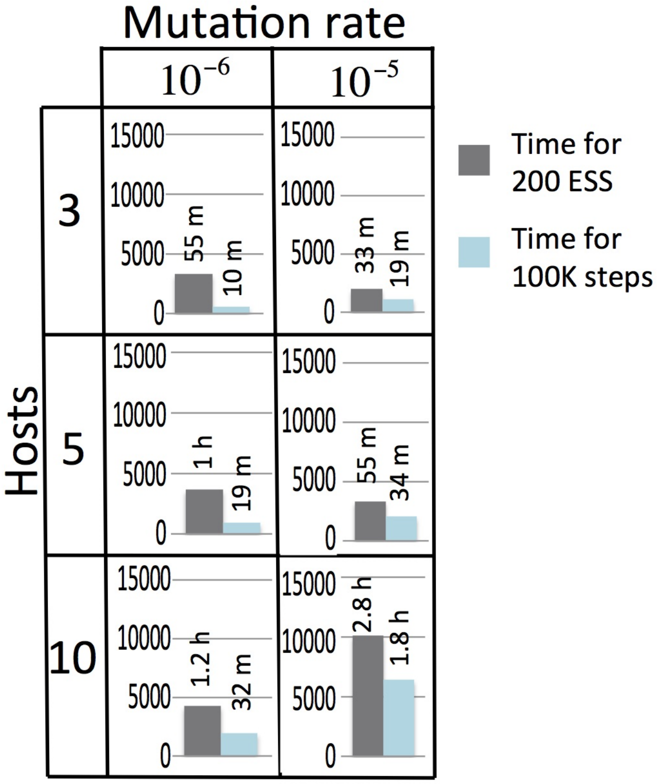

**Figure B. Inference of transmission in the early 2014 Ebola outbreak in Sierra Leone, only high-probability transmissions.** Transmission events with posterior probability higher than 5% as inferred by BadTrIP. The notation is as in Figure 5

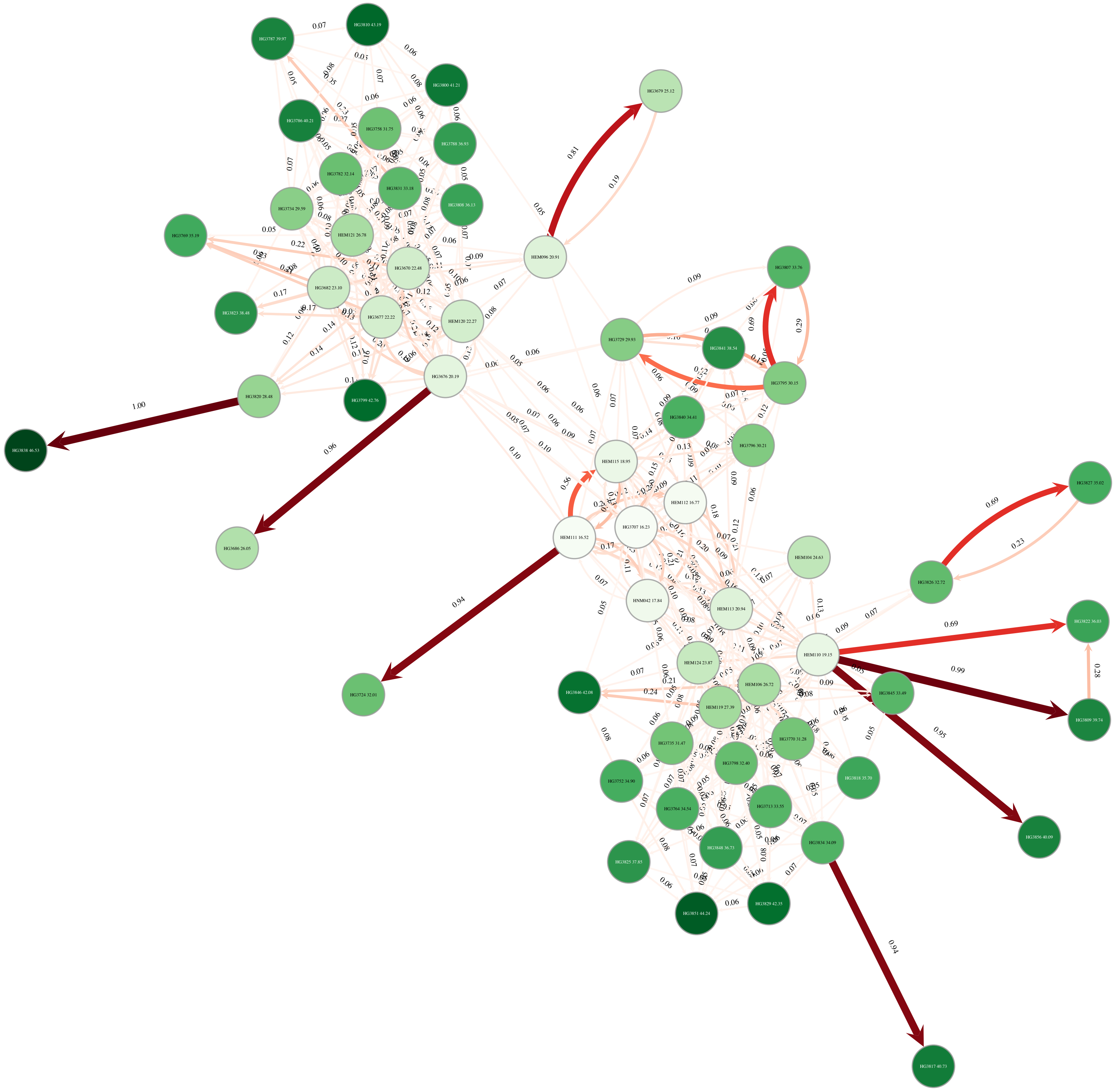

**Figure C. Graphical representation of the model parameters and transmission tree.** We again graphically represent three hosts in an outbreak using black rectangles: $H1$ infects $H2$, which in turn infects $H3$. Time is on the vertical axis, transmission bottlenecks are represented by blue dots, while sampling events are represented by black dots. $i_H1$ and $r_H1$ are the introduction and removal times of host $H1$, $t_{H1}$ is the infection time of host $H1$, and $I_{H1}$ is the infector of host $H1$ (and so on for the other hosts). There are 3 samples in this scenario, $S1$ (collected from $H1$), $S2$ (collected from $H3$), and $S3$ (also collected from $H3$), so $H3$ has 2 samples and $H2$ has none. For each sample we show the genetic data for the first position of the genome (for example, $G_{S2,1}$), which consists of 4 natural numbers, the 4 nucleotide counts at that position and sample (A's in green, C's in blue, G's in yellow, and T's in red). The names of model parameters that are inferred in BadTrIP are shown in green, while names of parameters that are fixed and are considered data are shown in black. The red line shows the transmission tree (or equivalently population tree). The tips of the tree are samples, but samples can also be internal nodes of the phylogeny with only one child. Transmission events correspond either to bifurcation nodes or to internal nodes with only one child. In this particular case the tree has only one tip, but in general it can have any number of tips.

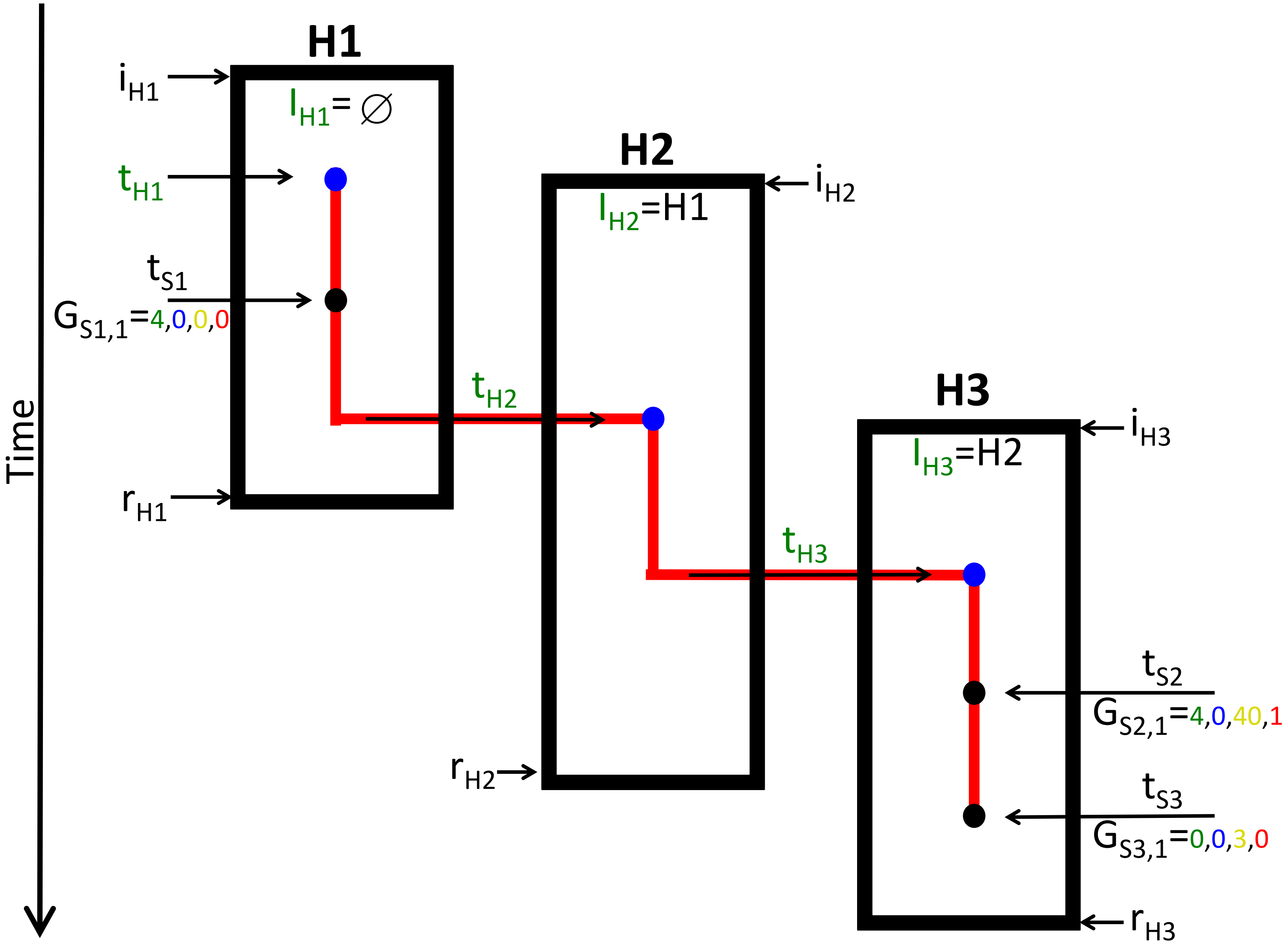

**Figure D. Graphical representation of the PoMo matrix $\boldsymbol{E}$.** $N$ is the virtual population size. We adopt an HKY [1] mutation model, with transition rate $\kappa$ and transversion rate $\mu$. $M^{i,j}$ represents the drift rate from count $i$ to $j$, which is $M^{i,i+1} = M^{i,i-1} = \frac{i}{N}\frac{N-i}{N}R$, for $i = 1, \ldots, N-1$, and $R$ being the time-scaled drift rate. The two columns on the left side of the matrices and on the row on top of them contain the states of the Markov model. Among these, A, C, G and T refer to the states in the virtual population when all individuals in the population have the same corresponding nucleotide. Instead $\binom{i,I}{N-i,J}$ represents the state when $i$ individuals in the virtual population have nucleotide $I$ and $N-i$ have nucleotide $J$. This is a re-adaptation from the equations in the supplementary material in [2]. Matrix $\boldsymbol{E}_D$ is obtained from $\boldsymbol{E}$ by setting the mutation rates to 0.

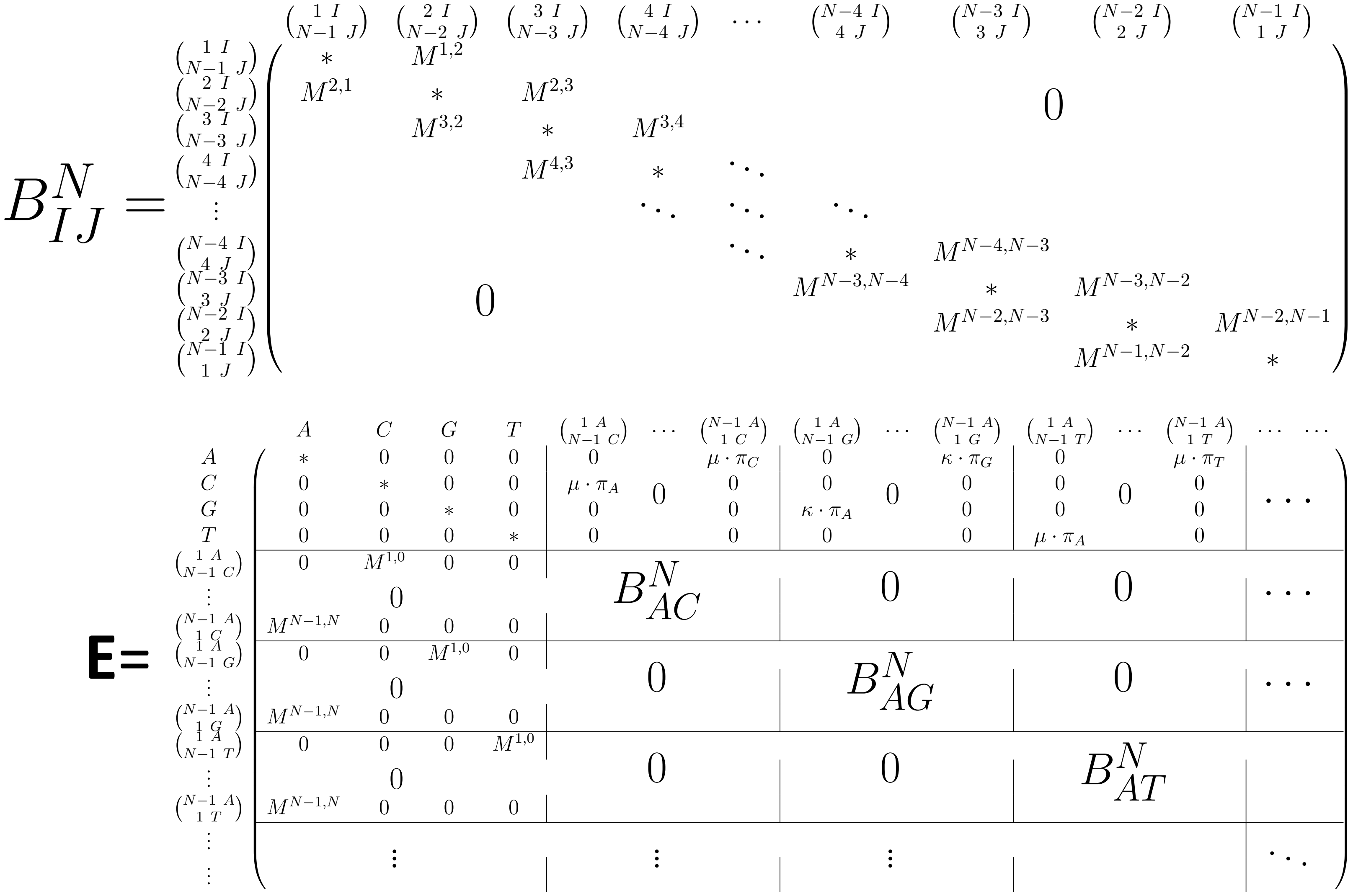

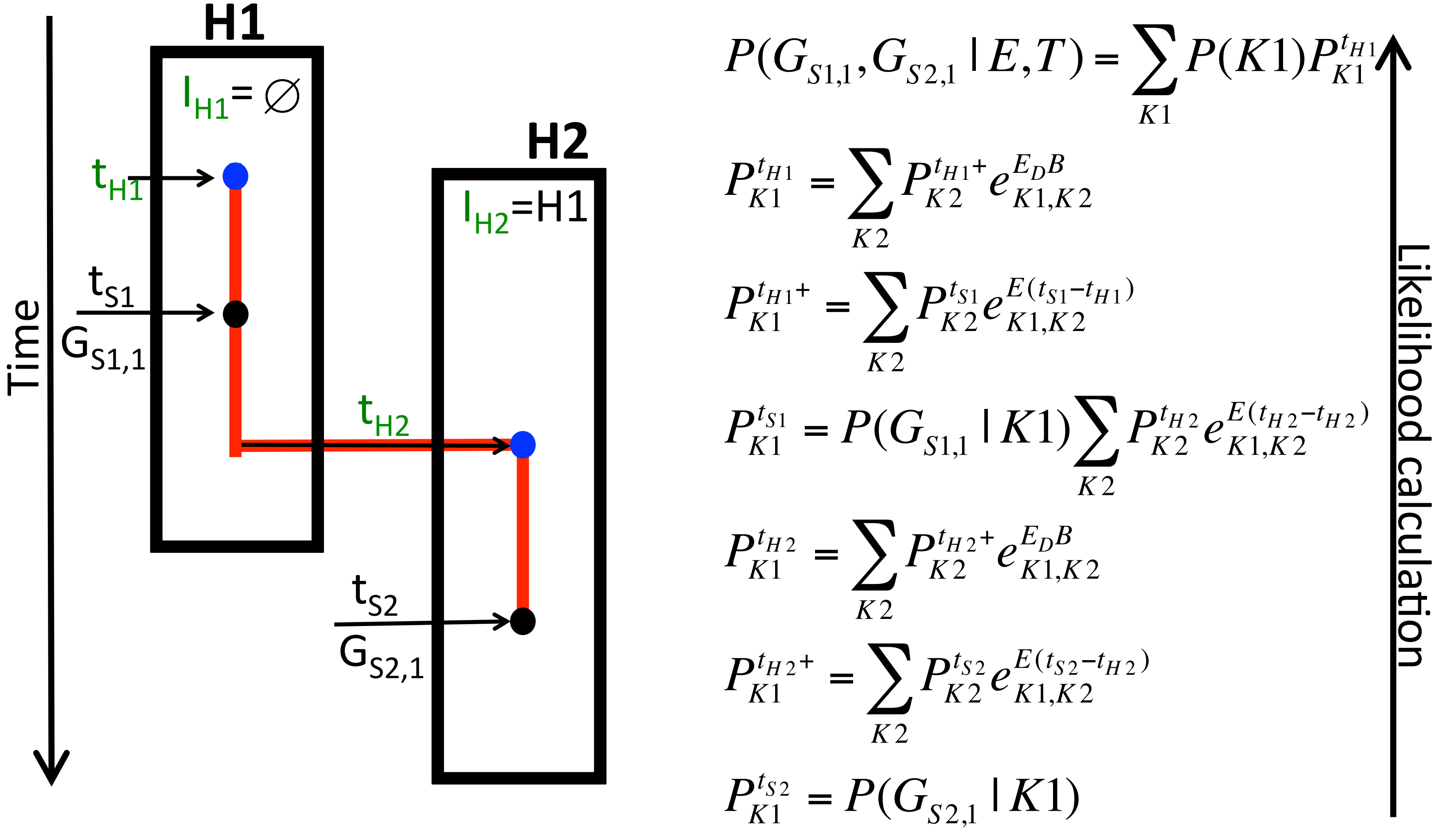


**Figure E. Graphical representation of likelihood calculation.** Here we call the genetic evolution matrix $\boldsymbol{E}$, and the tree $\boldsymbol{T}$ (red line). Parameters and graphics on the left side of the figure are the same as in Figure C. On the right side, the likelihood calculation starts at the bottom with the last sample collected, and is stopped when it reaches the top of the tree (the bottleneck of the transmission event of the index case). $P(G_{S,1}|K)$ correspnds to sampling events (black dots in the figure) and represents the likelihood of state $K$ given the first position of sampled data $S$ (here we only consider the first position of the genome for simplicity); this can be calculated using equation 2. $P_K^t$ represents the likelihood of state $K$ at time $t$ given all data below the considered node and within its sub-phylogeny. If $t$ is the time of a transmission, $t+$ represents the time right after (forward in time) the instantaneous transmission bottleneck, and $t$ the time right before it. $e_{K1,K2}^{E_D B}$ represent the probability changes due to the transmission bottleneck (blue dots in the figure). The final likelihood is obtained summing the likelihoods of all states at the root (time $t_{H1}$ in this case), each weighted by the prior probability $P(K)$ of the state (the latter is caculated by assuming equilibrium).

**Figure F. Posterior support for true infector in BadTrIP on simulated data.** **A**) Mean posterior probability of the true infector of each case (averaged over all cases and replicates). **B**) Frequency with which the correct transmission event has higher than 5% posterior probability. Bars represent percentages (from 0, worst, to 100, best) for BadTrIP (red), SCOTTI [3] (yellow) and the shared variants-based clustering (SVC) approach [4] (blue). On the x axis are different simulation scenarios with the first one, "base", being the basic simulation scenario with 10-15 cases per outbreak, about 300-500 SNPs among all hosts, recombination 10 times stronger than mutation, complete bottleneck (no transmission of within-host genetic variants), read coverage of 40x, PoMo virtual population size of 15, actual pathogen population size of 1000, and genome size of 5 kb. All other scenarios are obtained from the base one changing one or two parameters: in "no recombination" the recombination rate is set to 0; in "high recombination" the recombination rate is 10 times higher; in "high mutation" the mutation rate is 10 times higher resulting in 2000-3000 SNPs per outbreak; in "low mutation" the mutation rate is 10 times lower resulting in 30-50 SNPs per outbreak; in "very low mutation" the mutation rate is 1000 times lower, resulting in 0-1 SNPs per outbreak; in "weak bottleneck" at transmission 5 pathogen units from the infector colonised the infected host, instead of just 1; in "high rec. weak bott." both the recombination rate is 10 times higher and the founding population at transmission is made of 5 pathogen particles; in "high coverage" read coverage in sequencing is 100x instead of 40x; in "1x coverage" read coverage in sequencing is 1x instead of 40x; in "sequencing error" 0.2% of read bases are randomly modified to simulate sequencing error, coverage is reduced to 20x, and genome size is reduced to 1kb; in "high N" the PoMo virtual population size is 25 instead of 15.

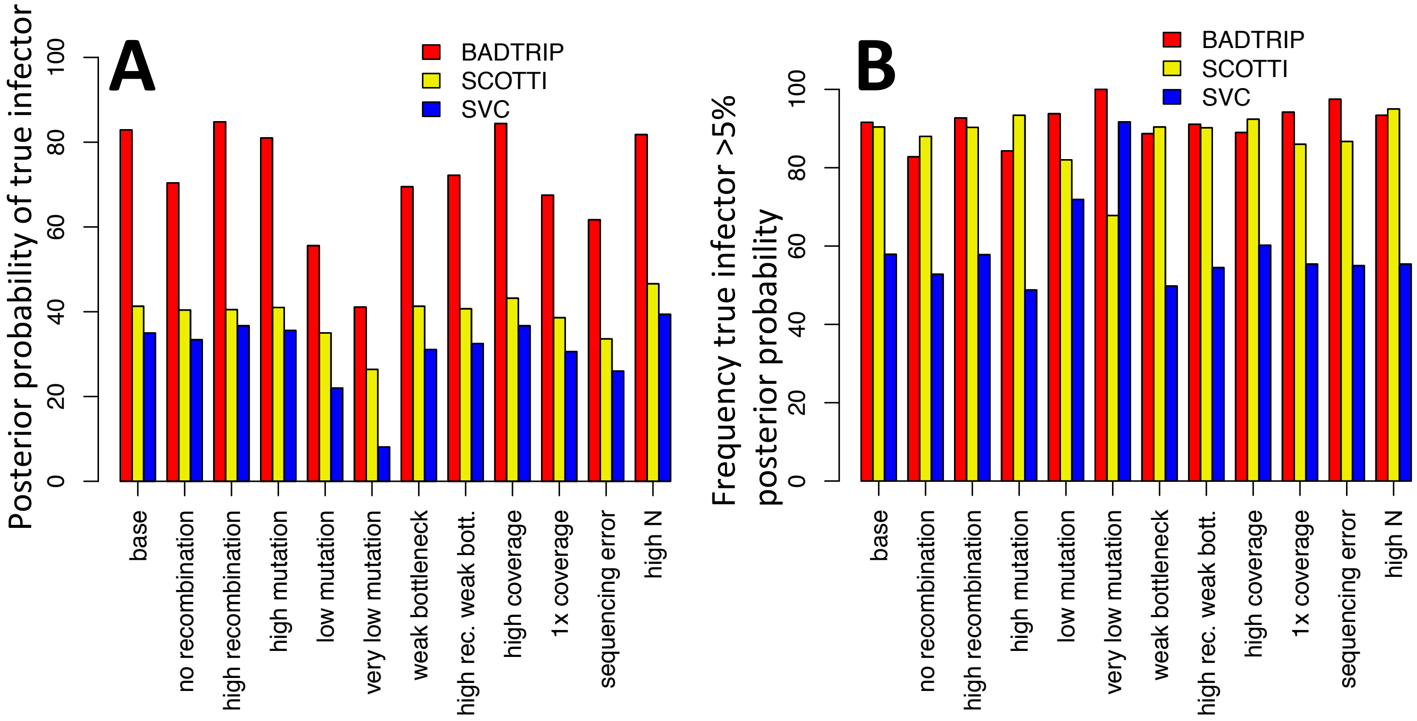

Supplement: S1 Text — (PDF) [file pcbi.1006117.s001.pdf]
